# Malnutrition in Older Adults—Recent Advances and Remaining Challenges

**DOI:** 10.3390/nu13082764

**Published:** 2021-08-12

**Authors:** Kristina Norman, Ulrike Haß, Matthias Pirlich

**Affiliations:** 1 Department of Geriatrics and Medical Gerontology, Charité–Universitätsmedizin Berlin, Corporate Member of Freie Universität Berlin and Humboldt-Universität zu Berlin, 13347 Berlin, Germany; 2 Department of Nutrition and Gerontology, German Institute of Human Nutrition Potsdam-Rehbrücke, 14458 Nuthetal, Germany; ulrike.hass@dife.de; 3 Institute of Nutritional Science, University of Potsdam, 14458 Nuthetal, Germany; 4 German Center for Cardiovascular Research (DZHK), Partner Site Berlin, 10115 Berlin, Germany; 5 Imperial Oak Outpatient Clinic, Endocrinology, Gastroenterology & Clinical Nutrition, 12159 Berlin, Germany; pirlich@kaisereiche.de

**Keywords:** malnutrition, ageing, inflammaging, sarcopenia, anorexia of aging, micronutrients, DoMAP, GLIM criteria

## Abstract

Malnutrition in older adults has been recognised as a challenging health concern associated with not only increased mortality and morbidity, but also with physical decline, which has wide ranging acute implications for activities of daily living and quality of life in general. Malnutrition is common and may also contribute to the development of the geriatric syndromes in older adults. Malnutrition in the old is reflected by either involuntary weight loss or low body mass index, but hidden deficiencies such as micronutrient deficiencies are more difficult to assess and therefore frequently overlooked in the community-dwelling old. In developed countries, the most cited cause of malnutrition is disease, as both acute and chronic disorders have the potential to result in or aggravate malnutrition. Therefore, as higher age is one risk factor for developing disease, older adults have the highest risk of being at nutritional risk or becoming malnourished. However, the aetiology of malnutrition is complex and multifactorial, and the development of malnutrition in the old is most likely also facilitated by ageing processes. This comprehensive narrative review summarizes current evidence on the prevalence and determinants of malnutrition in old adults spanning from age-related changes to disease-associated risk factors, and outlines remaining challenges in the understanding, identification as well as treatment of malnutrition, which in some cases may include targeted supplementation of macro- and/or micronutrients, when diet alone is not sufficient to meet age-specific requirements.

## 1. Introduction

The World Health Organization (WHO) has declared Healthy Ageing a priority of its work on ageing between 2016 and 2030 and developed a policy framework which emphasizes the need for action across multiple sectors [1]. The aim of the program is to enable older persons to develop and maintain functional ability which permits wellbeing and allows them to partake in society [1]. Older adults (individuals aged 65 or over) are the fastest growing age group and projections from the United Nations predict that by 2050, the number of adults aged 65 or over will be twice as big as the amount of children under the age of five and also surpass the number of adolescents aged between 15 and 24 years. In 2050, improvements in survival are expected to add approximately 5 years to the life expectancy at birth for the world’s population which was 72.6 years in 2019 [2].

Biology of ageing is understood as the time related decline of physiological functions, leading to changes in functional performance of different organ systems as well as with reduced resilience to physical, cognitive and mental stressors, however, there are great individual differences in these changes. Advanced age is associated with reduced adaptive and regenerative capacity which is leading to higher rates of morbidity [3]. On the other hand, the presence of age-associated diseases in middle-aged individuals has been interpreted as a sign of accelerated ageing [4]. Maintaining an adequate nutritional status as well as a sufficient nutrient intake is key to health and quality of life and as such is one prerequisite for wellbeing in higher age and modulator of healthy ageing as defined by the WHO. However, older adults are susceptible to nutritional problems and ultimately also to malnutrition through a variety of mechanisms [5]. As age is one main risk factor for the development of chronic disease, older persons are particularly susceptible to disease-related weight loss, loss of muscle mass and strength (i.e., sarcopenia) and ultimately, the frailty syndrome, all of which can fundamentally impact recovery from disease and clinical outcome in general [6,7,8]. Weight loss, a marker of macronutrient deficiency and/or catabolism, is a common key initial phenomenon in old patients, which sets off a catabolic cascade of unfavourable events resulting in higher morbidity and mortality. The causes for weight loss in higher age are multifactorial, but can in part be attributed to both disease processes such as catabolic events, disease or age-related anorexia (“anorexia of aging”) and subsequent insufficient dietary intake, but also to increased inflammatory status, depressive or cognitive disorders [9] as well as a decreased socio-economic status [10]. But even outside the context of disease or manifest disorders, both ageing processes and age-associated changes can slowly impact physiology and metabolism and thus effect a gradual change in the nutritional status of older adults [11].

The treatment of malnutrition requires early identification and multimodal intervention, in hospitalized patients as well as community dwelling older adults. However, treatment modality still poses a challenge for nutritional therapy with yet open questions [12,13]. This review summarizes the current state of evidence on the complex aetiology of malnutrition in old adults, considering both effects of ageing processes and disease-related factors. Also remaining challenges in the identification and treatment of malnutrition in the old are outlined.

## 2. Impact of Malnutrition in the Old

Although clinical malnutrition predominantly occurs in patients in hospitals, care situations or nursing homes, malnutrition, nutritional risk and specific nutrient deficiencies in particular, are a common albeit frequently overlooked occurrence in community-dwelling old people [14,15].

Consequences of malnutrition are deleterious and far reaching and have been described in detail [6]. While disease-related malnutrition is not limited to older adults, it is more frequent in higher age, and the consequences appear to be more severe in older persons due to their impaired regenerative capacity.

Malnutrition in general has serious implications for clinical outcome, for recovery from disease, trauma and surgery and is associated with increased morbidity and mortality both in acute and chronic disease [6] and has thus been acknowledged as serious burden for the health care system [16].

Depending on the type of malnutrition, protein catabolism can be pronounced. Disease-related malnutrition therefore leads to rapid skeletal muscle wasting, whereas age-related malnutrition is associated with a slower but progressive loss of muscle mass. The effects of protein catabolism are prominently reflected by lower muscle mass, muscle strength and function with severe implications for physical performance [17]. At the same time, malnutrition [18] but also reduced dietary protein intake per se [19] are associated with a decrease in bone mineral mass in higher age. Together with poor physical performance and coordination resulting in higher fall risk, these factors accelerate the age-related risk of osteoporosis and osteoporotic fractures [20]. Taken together, the risk of falling and the subsequent loss of independence and disability are greatly increased.

Malnutrition-related protein catabolism and micronutrient deficiency have been prominently linked to the impairment of immune function [21], which is already affected in higher age. In older malnourished adults, this manifests as the loss of cell-mediated immunity in particular [22], increasing the risk for infection and delaying recovery from disease [23]. Several studies have thus shown a close relationship between malnutrition and risk for infection, such as healthcare-associated infections [24], infectious complications and subsequent longer stays in intensive care units (ICU) and increased ICU mortality in older malnourished patients [25].

Wound healing as well as tissue recovery are also impaired in malnutrition, which in part can be attributed to micronutrient deficiency [26]. This clearly predisposes older adults to wound healing disorders and chronic wounds which are a great burden to patients as well as associated with decreased quality of life and furthermore with higher expenditure in the health care setting [27].

One particularity of malnutrition in older adults is that the impact appears to be more severe than in younger adults. Studies have not only shown that changes in body composition in malnutrition occur to a greater extent in older compared to younger adults [28], but also that recovery of low body cell mass or muscle mass is impaired in higher age following weight loss [17]. This predisposes malnourished older adults to the risk of developing the so-called geriatric syndromes which have been described as multifactorial syndromes typical in higher age [29]. Geriatric syndromes greatly compromise health status, cognitive functioning, functional ability, and compensatory capacity [29] and result in higher mortality, albeit predominantly in the younger old [30].

### 2.1. Role of Malnutrition in the Geriatric Syndromes Frailty, Fatigue, Sarcopenia

Malnutrition plays an important role in the development of certain geriatric syndromes. Geriatric syndromes are complex multifactorial conditions occurring in higher age with serious implications for health [29] and have been described as “phonotypical presentations of accumulated and underlying ageing-related dysfunctions spanning over different organ systems” [31]. They include (but are not limited to) dementia and delirium, depression, incontinence, fall risk, visual as well as hearing impairment, wound healing disorders, frailty, and sarcopenia [32].

Involuntary weight loss, a hallmark of malnutrition, is inevitably associated with loss of skeletal muscle mass, which appears to occur at a greater extent in higher age. This increases the risk of developing sarcopenia, a phenomenon which is characterized by the loss of both muscle mass as well as muscle strength and function. As these two entities frequently occur together, this has led to the new term “sarcopenia malnutrition syndrome” [33] and a need for new screening tools which reliably identify both conditions has been voiced [34].

A phenomenon, which is often overseen in this content but nonetheless is important in an increasingly obese society, is sarcopenic obesity [35], which describes a condition with reduced lean mass and increased fat mass, resulting in a high-risk body composition phenotype. This underlines the importance of evaluating muscle mass independently from weight loss, since age-related changes in body composition such as increases in (visceral) fat mass may well mask low lean mass. Furthermore, ectopic fat infiltration in the muscle lowers the quality of skeletal muscle and thereby impairs muscle functionality [35].

As sarcopenia has been called the biological substrate of frailty, the close relationship between malnutrition and sarcopenia suggests a link between malnutrition and frailty as well. Not surprisingly, involuntary weight loss, which is an indicator of catabolism, is a major risk factor for developing physical frailty [36]. Weight loss is therefore not only one of five factors such as fatigue, weakness, slow gait speed and low physical activity which constitute frailty as defined by the frailty phenotype [37], but is also linked causally to the other four factors [36]. As such, there is a close relationship between malnutrition and frailty which has been well documented [38], although they are considered conceptually distinct conditions. Numerous studies have shown a significant overlap of both entities in hospital patients [39] and community dwelling old [40,41]. There is even evidence that the consequences frequently attributed to malnutrition might in reality be due to the effects of frailty. An evaluation in 2804 community-dwelling older adults from the Singapore Longitudinal Aging Study II revealed that functional decline measured by decreased (instrumental) activities of daily living, disability, impaired quality of life and long-term mortality in particular was more apparent in older adults with physical frailty with or without malnutrition [42].

Moreover, fatigue is also one of the core elements of frailty. Fatigue has been described as a relentless exhaustion affecting the ability to carry out physical and mental activities [43] and has been linked to age-related mitochondrial dysfunction [44,45]. Old patients with severe involuntary weight loss at discharge from hospital had a significantly higher risk for severe fatigue which in turn compromises post hospital recovery [46]. Nutritional status including both malnutrition and obesity, has moreover been identified as an important modulator of fatigue [47], and more evidence is warranted on the role of dietary approaches, including anti-inflammatory diets, in the treatment of fatigue [48].

Due to the proposed relationship between long-term dietary patterns and cognitive function, malnutrition has also been linked to cognitive impairment [49,50], although the relationship is complex and difficult to disentangle and more studies are needed on this subject [50]. Similarly, there is a close interaction between malnutrition and depression [51,52,53], but causality is difficult to establish, as the relation is most likely mutual.

### 2.2. Prognostic Impact of Malnutrition in the Old on Mortality

It is well established that malnutrition is associated with increased mortality in both acute and chronic disease, and this effect is still observed in older adults. The risk for both short-term mortality in acute conditions [54] as well as for long-term mortality in chronic disease is significantly increased [55]. One large cohort study included 1767 older individuals with a variety of disease ranging from cancer to diseases of the circulatory or respiratory system and revealed that the increased risk of mortality due to malnutrition existed irrespective of the cause of death [56].

## 3. Malnutrition: Definition and Types and How to Screen for Them

Despite an ongoing debate, there is still no universally accepted definition of malnutrition [57]. Therefore, in 2016, the world’s four leading Clinical Nutrition Societies (ESPEN, ASPEN, FELANPE, and PENSA) representing more than 70 national scientific societies started a consensus process in order to develop criteria for malnutrition which could be used in all clinical settings on a global scale. The resulting concept of the Global Leadership Initiative on Malnutrition (GLIM) [58,59] was published in 2019 and considers three phenotypic criteria for the diagnosis of malnutrition: weight loss (>5% within past 6 months, or >10% beyond 6 months), low body mass index (BMI) (<20 kg/m^2^ if <70 years, or <22 kg/m^2^ if >70 years), reduced muscle mass (according to validated body composition techniques), and two etiologic criteria: reduced food intake or assimilation (≤50% of energy requirements >1 week, or any reduction for >2 weeks, or any chronic gastro-intestinal condition that adversely impacts food assimilation or absorption), and inflammation (acute disease/injury or chronic disease-related). It is proposed that the diagnosis of malnutrition is based upon the presence of at least one phenotypic and one etiologic criterion, and in a second step different thresholds of the criteria can be used for severity grading of malnutrition. The GLIM criteria are subject of numerous ongoing validation studies. A recently published study on community-dwelling older adults participating in a long-term osteoporosis trial in Hongkong demonstrated that the GLIM criteria were associated with a higher risk for sarcopenia, frailty, and mortality during a 14-year follow-up period [60]. While the GLIM criteria are not age-specific, they do include age as a risk factor among the components. For geriatric patients, the guidelines on Enteral Nutrition in Geriatrics by the European Society of Clinical Nutrition and Metabolism (ESPEN), have defined clinical malnutrition as the presence of either weight loss which reflects a catabolic state (>5% in six months) and/or low BMI (i.e., BMI below 20 kg/m²) which represents depleted physiological stores [61].

Nutritional risk is less well defined but commonly understood to be a condition in which the present nutritional status is at imminent risk of impairment due to a range of factors such as medical history, comorbidities or drugs which might increase dietary requirements or interfere with nutrient absorption or metabolism. Further factors may include physical, mental or cognitive status which might prevent the older person to properly care for themselves as well as socio-economic factors which hinder access to a varied high-quality diet [61].

Screening tools which are suitable for the use in older adults need to focus on the most important risk factors for malnutrition in high age, in order not only to diagnose manifest malnutrition but also to capture the risk to develop malnutrition as described above. That way, screening tools can be used to identify patients early in order to initiate nutritional treatment. Several screening tools have been established for the specific use in older adults, but the most widely used and most studied instrument is the Mini Nutritional Assessment (MNA) in its long or short form. However, due to its broad range of covered topics, the specificity of the MNA has been questioned, as it is associated with a high risk of “over-diagnosing” malnutrition in the old [62]. This problem might be countered by complementing the MNA with the GLIM criteria as suggested [59].

Screening for and treating malnutrition has also been acknowledged as one of the first necessary steps in the identification and treatment of sarcopenia [63]. Recent studies have compared the ability of screening tools for malnutrition such as the MNA short or long form to predict the development of sarcopenia to newer diagnostic tools such as the GLIM criteria [58,59]. In the SarcoPhAge cohort, the GLIM criteria did predict incident sarcopenia, whereas neither of the MNA forms did [17].

### 3.1. Macronutrient Deficiencies

Clinical malnutrition results from an imbalance between macronutrient intake and requirement [64], which causes a measurable reduction in tissue and ultimately weight. Due to the frequently insufficient protein and energy intake, it is therefore also commonly referred to as protein-energy malnutrition (PEM) or protein-energy undernutrition (PEU). Consequently, there is concomitant need for both adequate energy intake (25–30 kcal/kg body weight, depending on individual situation) and higher protein intake [65]. There is overwhelming evidence that protein requirements are generally higher in older age (1.0–1.2 g/kg body weight) and adequate protein intake [65,66] is crucial in order to prevent malnutrition and sarcopenia. Recommendations regarding protein intake are mainly based on the concept of anabolic resistance, which describes an impaired capacity of the muscle to respond to anabolic stimuli in higher age. However, protein intake in community-dwelling older adults has been reported to be frequently well below recommended intake [67,68] which is associated with a higher risk for the development of malnutrition.

Also, depending on the type of malnutrition and underlying disease, protein catabolism can be pronounced. Protein requirements are therefore further increased in older adults with malnutrition or disease (1.2–1.5 g/kg body weight) [65,66].

### 3.2. Micronutrient Deficiencies

One specific form of malnutrition frequent in older adults are micronutrient deficiencies [10,69]. In contrast to quantitative malnutrition, which is reflected by weight loss, micronutrient deficiencies are much harder to screen for and identify, in part also due to methodological issues such as missing suitable markers of stored or available micronutrients. The majority of dietary intake surveys have, however, identified an inadequate intake of a broad range of micronutrients in older adults [70]. Since micronutrient deficiency such as iron (Fe), vitamins C and D, vitamins B6 and B12, as well as folic acid and the trace element zinc (Zn), have been prominently linked to the impairment of immune function [71], the following chapter focusses on recent findings regarding these nutrients in particular.

The reasons for insufficient micronutrient intake vary including not only a low amount of food, but typically also the choice of food and the lack of variety. Prices, availability of foods rich in vitamins, trace elements and minerals can reduce the intake of micronutrients. On the other hand, ageing is also associated with changes that facilitate deficiencies in calcium, vitamin D, vitamin B12, Fe, magnesium, and Zn among other important nutrients. Large studies on the micronutrient serum status in older adults are rare due to the costs and efforts. The Population Based KORA-Age Study, a representative cohort study, recently showed subclinical micronutrient deficiencies in community-dwelling old [69]. 52.0% of the 1079 older study participants had a vitamin D deficiency (<50 nmol/L), 27.3% had low vitamin B12 levels (<221 pmol/L), 11.0% had insufficient Fe levels (men <11.6 µmol/L, women <9.0 µmol/L), and 8.7% had low folate levels (<13.6 nmol/L). Among the risk factors for the subclinical deficiencies were advanced age, frailty, lack of physical activity and no or irregular use of dietary supplements. Dietary supplement intake is an easily modifiable risk factor, and recent studies have confirmed that regular use of dietary supplements is associated with lower rates of subclinical deficiencies of nearly every micronutrient investigated in the study [72]. Age-specific requirements are not really clear, but higher intakes have been recommended due to the frequently impaired intestinal absorption or distribution [73]. Also, it has recently been postulated that diet alone might not be enough to meet the age-specific requirements of older adults, but tailored supplementation of micronutrients might well be necessary [74], in order to e.g., support the immune function. The intake of drugs may also interfere with the metabolism of micronutrients [75,76] which further compounds the problem as poly-pharmacy is common in higher age.

Moreover, it is known that inflammation, which is common in older adults, affects trace element status and their biomarkers, resulting in depleted plasma stores of Fe, Zn, and manganese, a phenomenon which is partly known as nutritional immunity and serves as a defence against invading pathogens [77].

Due to the multifactorial role of micronutrients in various metabolic processes as well as immune functioning, cell proliferation and growth, signalling processes and genomic stability, micronutrient deficiencies are in turn involved in the pathogenesis of a variety of conditions and age-related diseases [78]. One of the most well studied trace elements in higher age is Zn, and dietary Zn intake has also been reported to be frequently insufficient in older adults [78]. Age-associated alterations of intestinal absorption, problems regarding chewing and swallowing, drug-interactions, and impaired subcellular processes in Zn metabolism can further contribute to low Zn absorption and availability. In turn, low dietary Zn intake with subsequent Zn deficiency has been linked to depressive disorders [79], loss of appetite and cachexia in age-advanced old [80], and to increased muscle catabolism via inflammatory cytokine activation [81] as well as to immunosenescence [82] and frailty [83]. Moreover, in older adults, an inadequate intake of antioxidant micronutrients such as vitamin E, carotenoids, and vitamin C has also been linked to the development of impaired muscle strength and physical performance [84,85,86,87].

## 4. Prevalence of Malnutrition

Despite the body of evidence describing the personal and clinical consequences of malnutrition and its economic impact on the health care system, malnutrition in the old remains a considerable problem with reported high frequencies, especially in situations of dependency [88]. This has been attributed to poor awareness and lack of time or education in medical as well as nursing staff, but recognition and treatment of malnutrition in older adults is undeniably a challenge even when identified early. All in all, it is estimated that roughly a quarter of European adults over the age of 65 are at high risk of malnutrition across various settings [89,90].

Prevalence of malnutrition, however, strongly depends on the setting, on underlying or accompanying diseases as well as on screening and assessment methods. Numerous studies have investigated prevalence of malnutrition in hospital and nursing home settings; but recently, several meta-analyses have been published which also use meta-regression to further explore determinants. Although the specific number of malnutrition prevalence differs between meta-analyses, the main findings are comparable, showing the lowest percentage of malnourished individuals in the community setting and the highest percentage in acute and subacute care settings, higher prevalence rates in higher age as well as sex-specific differences, as women had the highest risk. Also, region and instrument used to identify malnutrition had a significant impact on prevalence. One systematic review and meta-analysis of prevalence data of malnutrition and nutritional risk in older adults across different healthcare settings showed a wide range of malnutrition from 3% in the community setting to approximately 30% in rehabilitation and subacute care, even though the review only included studies using the MNA [91]. This study was recently complemented by two systematic reviews and meta-analyses [89,90]. Leji-Halfwerk et al. included further studies using 22 malnutrition screening tools validated for use in adults aged 65 years or more. They reported pooled prevalence rates of high malnutrition risk across all countries and screening tools which ranged from 8.5% in the community setting to 28.0% for the hospital [89,90]. The meta-analysis and systematic review by Crichton and colleagues focussed on studies carried out in community-dwelling older adults who had been assessed with Subjective Global Assessment (SGA), Patient-generated (PG)-SGA or MNA and found clear differences across countries with low prevalence in Northern Europe and highest in Southeast Asia. Using meta-regression, both meta-analyses reported higher prevalence rates in adults aged above the age of 80 [89,90], in women, in patients with one or more comorbidities and a higher prevalence of malnutrition in rural rather than metropolitan regions [90].

Recent advances in prevalence research has also included re-evaluation of large pool datasets using harmonized definitions of malnutrition indicators such as low BMI, weight loss, low food intake and combinations of these. Wolters et al. were able to show varying prevalence of malnutrition according to the different criteria used and concluded that it might be more useful to consider the criteria separately as each may reflect a distinct nutritional problem [92].

## 5. Determinants of Malnutrition

Malnutrition in older adults is of complex and multifactorial origin. A variety of factors such as life-style factors, disease and ageing processes may be involved and interaction between these factors is common. Understanding risk factors is crucial in order to address malnutrition effectively, but the complex aetiology of malnutrition is still not perfectly understood.

In industrial countries, disease is one of the most common reasons for developing malnutrition and the onset of malnutrition can be both acute and slow. Age in itself is an established non-modifiable risk factor for malnutrition. Higher age is associated with physiological changes which can potentially slowly result in or further malnutrition such as impaired taste and smell, decreased gastric flexibility, reduced appetite, etc. As higher age clearly increases the risk for disease, there is considerable risk for potentiation of nutritional problems.

### 5.1. Modifiable Determinants of Malnutrition

Recent research has particularly focussed on the evidence of potentially modifiable risk factors of clinical malnutrition, as targeting these risk factors is a fundamental element in the prevention and treatment of malnutrition. A recent meta-analysis investigated which factors are associated with incident malnutrition and identified marital situation, hospitalisation and physical limitations as the most important predictors [93]. Another systematic review categorized the evidence grade of thirty potentially modifiable factors which have been associated with malnutrition; however, stating that robust evidence was lacking for most of the studied determinants and highlighting the need for high quality prospective studies [94].

In a modified Delphi process, an international expert group therefore developed a model for the theoretical framework on the aetiology of malnutrition termed DoMAP (Determinants of Malnutrition in Aged Persons) and specifically, potential causative mechanisms [95]. The model construct consists of three triangle-shaped levels illustrates the suggested direction of causality of the various risk factors for developing malnutrition. Malnutrition is at the inner core of the DoMAP model surrounded by different layers of risk factors. The immediate layer consists of the three principal conditions which result in malnutrition (low intake, increased requirements, and impaired nutrient bioavailability). The next layer consists of the factors which are believed to directly cause one of these conditions and the outermost level presents factors which impact the direct factors and indirectly cause one of the three main conditions. By illustrating the sequence of events which can cause malnutrition the DoMAP model offers a better understanding of the aetiology which can be used both in research as well as in clinical routine. By addressing factors, which can potentially impact the three principal components and thus cause malnutrition, the model also offers the possibility of early identification of patients at risk for malnutrition.

### 5.2. Age-Associated Changes as Risk Factors

Physiological factors which may precipitate malnutrition in higher age include sensory impairment such as diminished taste or olfactory dysfunction, delayed gastric emptying, and disturbed motility leading to a functional decline of the ageing gut [96]. Ageing is therefore also associated with an increase in colonic transit time, increased intestinal permeability, and, ultimately, altered intestinal microbiota [97], which includes loss of biodiversity, enrichment in opportunistic pathogens, and concomitant reduction of health-associated species, such as short chain fatty acid producing species [98]. The changes in the microbiome have recently been implicated in the development of loss of appetite and frailty and as such are also potentially promoting malnutrition [99,100], but more studies are needed in this context.

Moreover, a decrease in gastrointestinal hormones (e.g., ghrelin), with concomitant adverse changes in anorectic signalling (e.g., neuropeptide Y, peptide YY (PYY), orexin A, leptin, cholecystokinin (CCK)) [101,102,103] leading to an altered appetite regulation in higher age have been described. While ghrelin is the only appetite-enhancing peptide, other hormones such as CCK and PYY for instance are recognized as relevant mediators in satiety. Those gut-derived peripheral hormones as well as systemic insulin, glucose and fatty acid levels regulate central appetite and hunger in the hypothalamic region in a feedback loop [104,105,106]. As has been shown in a postprandial setting with 14 frail and 20 non-frail old (>70 years) as well as 19 young adults (20–65 years), older persons have higher levels of fasting insulin and glucose as well as increased and prolonged postprandial insulin, glucose and CCK levels compared to younger adults [107]. Furthermore, frail older persons exhibited significantly less hunger in the fasting condition and impaired gastric emptying and gallbladder contraction in the postprandial period [107]. Similarly, in a small study, the suppressed postprandial hunger in the old was paradoxically accompanied by sustained ghrelin levels [108].

Last but not least, in the case of reduced smell and taste, the reward system in the central nervous system (CNS) is hampered in the perception of pleasure associated with palatable foods [109] which may in turn further decrease dietary intake.

Some of the pathway dysregulations can be attributed to the chronically elevated inflammation observed in higher age since enhanced cytokine levels (e.g., tumour necrosis factor alpha (TNF-α), interleukin (IL)-1β) are able to affect appetite regions in the CNS [103,110]. The resulting loss of appetite and early satiety seen in older adults has been termed “anorexia of aging” and leads to an insufficient food intake with a higher risk for both quantitative and qualitative malnutrition [111,112]. One risk factor for anorexia in healthy older adults is, not surprisingly, ageing itself [113]. Furthermore, body weight dissatisfaction, which might lead to avid dieting, weight loss and eating disorders, is frequent in older adults [114] and has been studied as possible precursor of appetite loss. Ultimately, anorexia of ageing has even been proposed to be a geriatric syndrome [115] as it significantly and independently affects nutritional und functional status in the old [116,117].

Postprandial regulation in older adults has gained increasing attention in the last decade, especially with regard to muscle protein synthesis. In the old, a blunted postprandial muscle protein synthesis in response to dietary protein has been described [118,119,120], whereas the basal muscle protein synthesis does not appear to be affected [121]. Moreover, the lower protein balance response to hyperinsulinemia in older adults indicates insulin resistance of protein metabolism [122]. The so-called anabolic resistance, i.e., an impaired capacity of the muscle to respond to anabolic stimuli (such as dietary protein and resistance exercise) seen in higher age serves as one explanation leading to a slow onset of sarcopenia [123].

Recent research has also focussed on postprandial regulation of metabolic parameters. Age-related changes in postprandial glucose and insulin have been well established [107,124], but more recently, age-specific changes in the postprandial dynamics of fibroblast growth factor 21 (FGF21) have also been shown, resulting in considerably higher values in older compared to younger adults [125]. FGF21 is an important metabolic parameter which regulates glucose and lipid metabolism, but higher levels in older adults are paradoxically associated with higher mortality [126] and have been implicated in the loss of muscle [127,128] and bone mass [129] as well as in the cachexia anorexia syndrome in old hospitalized patients [130]. Altered postprandial regulation of FGF21 might contribute to explain the higher values. Similarly, concentrations of adiponectin, a mediator of FGF21 functions increase with age [131] and are also associated with all-cause mortality in the old [132]. Again, alterations in postprandial adiponectin response have been shown in higher age which were positively associated with the FGF21 response [133].

Taken together, in contrast to disease-related mechanisms such as catabolism, which can result in acute weight loss and malnutrition, altered appetite regulation in higher age and changes in postprandial metabolism are associated with gradual changes that may not necessarily result in manifest malnutrition but contribute to or exacerbate existent nutritional problems and facilitate nutritional deficiencies.

### 5.3. Inflammaging as a Risk Factor for Malnutrition

Under physiological conditions, inflammatory processes represent a desired, strictly controlled and usually self-limiting reaction. However, chronic inflammation has long-term negative effects on the entire system. Increasing knowledge indicates that ageing is accompanied by slightly but chronically elevated inflammation levels. This sterile and silent inflammation in old adults has been termed “inflammaging” [134]. Inflammaging has since been investigate a whole range of age-related diseases within the so-called “network theory of aging”. Recently, in the pandemic of coronavirus disease 2019 (COVID-19), inflammaging gained additional attention in view of the observed cytokine storm and autoimmunity during infection among older patients, which can result in multiple organ failure and is in general associated with a worse disease outcome [135,136] and of course potentially increases the risk for malnutrition, though data are lacking [137]. This severe systemic inundation of cytokines might be further triggered by the dysregulated immune system in old age (immunosenescence), which is expressed in an imbalanced homeostasis of pro- and anti-inflammatory mediators [135].

Although underlying mechanisms are still not fully elucidated, inflammaging has been characterized as a chronic low-grade inflammatory status, which not only is reflected by higher pro-inflammatory levels, but by a remarkable imbalanced cytokine network [138,139]. Research in centenarians has shown that they have less burden of the typical age-related (co-) morbidities and that those “long-livers” have better immunological coping strategies which are attributed to a balanced and more anti-inflammatory system [4,139]. The senescence-associated secretory phenotype (SASP) is considered to support key cellular processes in inflammaging, since the secretion of those pro-inflammatory mediators do not only disrupt local tissue structures and functions, but primarily perpetuate the vicious inflammatory circle. Further, accumulation of waste products or inadequate elimination of cellular damage, as well as age-associated mitochondrial damages that result in a higher release of reactive oxygen species and extracellular release of mitochondrial DNA in particular, belong to the damage-associated molecular pattern which activates the innate immune system [140,141].

Meanwhile, it is widely recognized that inflammaging is partly responsible for triggering many age-related diseases, e.g., Alzheimer’s Disease (“neuroinflammation”) [142], atherosclerosis and cardiovascular events [143,144], type 2 diabetes mellitus (“metaflammation”) [145], (osteo-) sarcopenia and frailty [146,147] as well as cancer [148,149,150]. Therefore, there is considerable potential that inflammaging also contributes to an impaired nutritional status [151], but more studies are needed to explore the relationship. In the old, increased levels of pro-inflammatory cytokines have early on been associated with cachexia (“geriatric cachexia”) [152]. Cachexia is a well described complex wasting syndrome which is driven by inflammation and reflected by a striking loss of skeletal muscle mass and function [153]. It is the most pronounced condition within the disease-related PEM spectrum and occurs in catabolic disease such as cancer, chronic kidney disease, HIV/AIDS, chronic obstructive pulmonary disease. Pro-inflammatory cytokines, such as TNF-α, initially called “cachectin” [154] and IL-6, also known as the “cytokine for gerontologists” [155,156], play a dominant role in this phenomenon. The underlying inflammatory processes actively fuel the muscle protein breakdown via pro-inflammatory cytokines (TNF-α, IL-6, IL-1β) which on the one hand stimulate the ubiquitin-proteasome signalling pathway, while on the other hand inhibiting anabolic pathways [157]. Although cachexia is generally associated with severe disease, there is definite overlap in the mechanisms that have been used to explain malnutrition in the old caused by inflammaging [158] and in geriatric cachexia [152]. Inflammatory and related immunological processes in older persons is clearly associated with a higher risk for malnutrition (as indicated by the MNA) [158].

**Figure 1:**
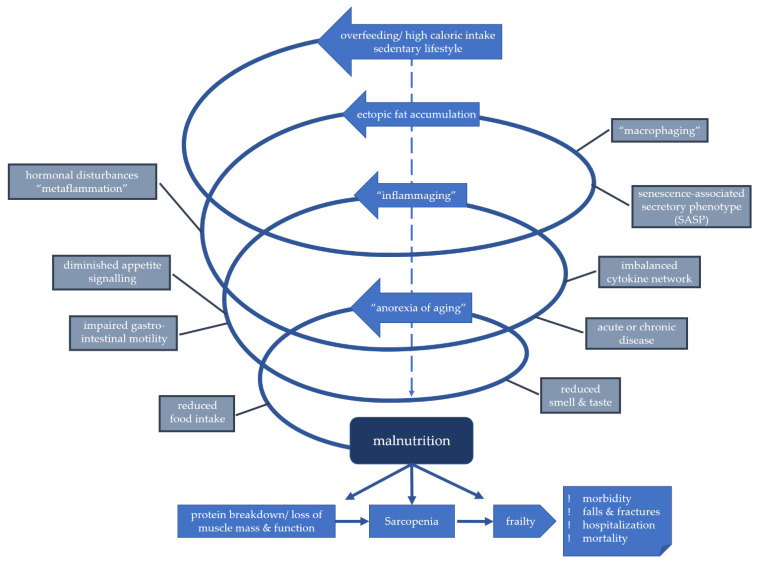
Model of downward spiral in the development of malnutrition in higher age.

Taken together, the aetiology of malnutrition is complex, and a multitude of risk factors can contribute to or aggravate the development of malnutrition as illustrated in Figure 1. While it is important to further understand age-associated changes which impact nutritional intake, nutrient absorption, digestion and metabolism, many of these factors are not easily identified or modified. They, however, need to be accounted for when assessing the risk for malnutrition and in the concept of treatment.

## 6. Treatment of Malnutrition

Acknowledging the different and complex risk factors, which can result in or aggravate malnutrition or contribute to the risk of developing it, it becomes clear that treatment of malnutrition is as complicated and challenging. Dependent on both setting and the situation of the older adult, different therapy approaches are warranted.

Regarding the overwhelming evidence that protein requirements are higher in older age [65,66], adequate protein with appropriate energy intake is crucial in order to prevent malnutrition and sarcopenia. Further, due to the frequent micronutrient deficiencies in higher age, targeted supplementation of micronutrients might be useful, when diet alone is not sufficient to meet the age-specific requirements. However, there are still considerable gaps concerning the evidence of non-pharmacological treatment of malnutrition.

Available studies so far have delivered conflicting evidence on the benefits of nutritional theory. Recently, an international panel of nutrition experts identified the shortcomings of these studies which include heterogeneous study populations and too short or varying treatment duration as well as issues relating study methodology such as inadequately described control group and no placebo, no blinding of study personnel, frequently no intention-to-treat-analyses as well as selective outcome presentation [13]. Therefore, it remains unresolved which interventions are most effective in which patient groups and whether specific approaches are needed depending on the aetiology of malnutrition [13].

A recent meta-analysis, which did not find convincing evidence of the use of oral nutritional supplements in the treatment of malnutrition in older patients, also highlighted the need for more and larger trials to identify suitable and effective interventions for treating malnutrition [159]. Pooling data from 9 randomized controlled trials from different care settings [160], Reinders and colleagues found evidence that dietary counselling with or without oral nutritional supplements was more effective across the settings than oral nutritional supplements alone, but the outcome parameters included merely energy intake and body weight, and no other relevant outcome parameters such as improved muscle strength or function were considered.

## 7. Remaining Challenges

**Figure 2:**
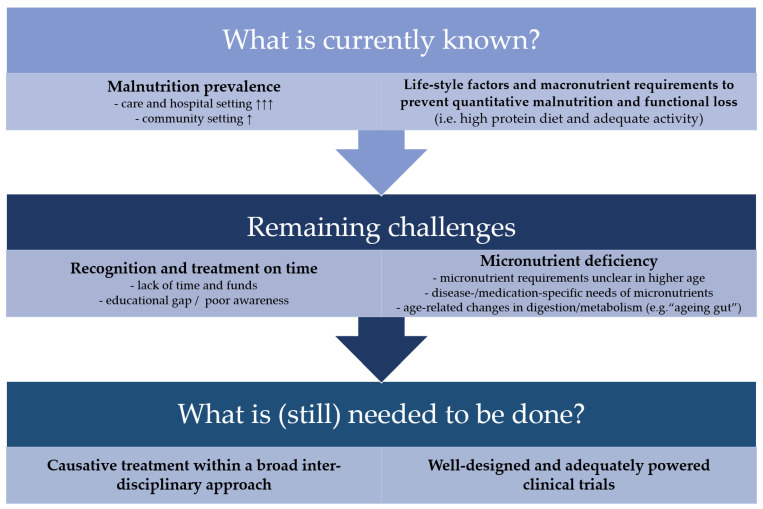
Remaining challenges in the effective prevention and sustainable treatment of malnutrition in old adults. ↑↑↑ indicates a high prevalence, whereas ↑ indicates a lower prevalence.

Despite the overwhelming evidence regarding the negative outcome of malnourished older adults, there are still many remaining challenges in the understanding, identification and treatment of malnutrition in older adults. They concern both the prevention of slow onset age-associated malnutrition as well as the treatment of disease-related malnutrition. Figure 2 summarizes open questions and remaining challenges in this field.

One particular concern regarding the prevention of malnutrition is that some nutritional requirements are not well established in higher age. Micronutrient and trace element concentrations have been shown to change with age [74] and have even been implicated to play a role in the ageing processes [161]. Changes in body composition and physical activity may lower energy requirements, but not the requirements of nutrients in general. In order to ensure a sufficient supply of essential nutrients, this requires a high nutrient density in the diet. Therefore, requirements of micronutrients which are frequently deficient in older adults, clearly need elucidation given their multifactorial role in promoting health. Also, while the need for some nutrients may change in higher age, ageing processes themselves may impact the requirements of certain nutrients, in particular in the presence of inflammation. As inflammation alters the trace element profile (increasing copper, decreasing selenium and Zn among other trace elements), an inflammation adapted intake of these trace elements may be necessary. However, more research is needed to elucidate inflammation related needs of the older population and the possible benefit of a tailored supplementation. Furthermore, as higher age is frequently associated with disease, disease-specific nutrient requirements may further complicate the picture.

Taken together, a need to critically appraise current nutrient recommendations for the older age group has been identified, and the WHO has been challenged to issue guidelines [162]. There is also an increasing demand worldwide for WHO guidelines which competent national authorities can use to address the nutritional needs of their growing older populations [70].

Moreover, although recent research on determinants of malnutrition has added considerable new evidence, the impact of ageing-related changes on the long-term development of malnutrition, still needs further elucidation. One example is the ageing gut and its altered microbiome, which have gained attention in the last years. Emerging technologies, such as e.g., high-throughput culturing, will further the research on ageing microbiome, so its role in the development of malnutrition might soon be elucidated. Novel dietary approaches which modulate the ageing microbiome such as the Mediterranean diet [163] are of interest in the prevention of malnutrition, and more studies are warranted. Considering the importance of muscle mass maintenance [164], nutritional intervention needs to focus on muscle mass and also, needs to be combined with exercise. Therefore, more studies on nutritional therapy together with different kinds of exercise regimen are clearly needed.

Lastly, not only the prevention of malnutrition, but also the topic of treatment of malnutrition needs more research, in terms of well-designed and adequately powered clinical trials in order to ensure sufficient statistical power to identify true treatment effects. Moreover, relevant outcome parameters need to be included, and a careful selection of target populations with well-defined malnutrition is necessary. Also, more research is needed for treatment approaches which specifically target the underlying causes of malnutrition themselves [165] i.e., causative treatment. This, however, not only requires a longer time frame and a broader inter-disciplinary approach, but more research regarding the most relevant causes and their common pathophysiology which would allow a “causation-oriented” multimodal treatment [13].

## 8. Conclusions

Taken together, more research is needed to understand which ageing-related changes are early predictors/precursors of malnutrition that in turn can be addressed in order to prevent the development of nutritional deficiencies. For clinically manifest malnutrition, more studies need to be performed in older adults in order to identify the suitable treatment for the various settings.

## Abbreviations

ASPEN	American Society for Parenteral and Enteral Nutrition
BMI	body mass index
CCK	cholecystokinin
CNS	central nervous system
COVID-19	coronavirus disease 2019
DoMAP	Determinants of Malnutrition in Aged Persons
DNA	deoxyribonucleic acid
ESPEN	European Society of Clinical Nutrition and Metabolism
Fe	iron
FELANPE	Federación Latinoamericana de Terapia Nutricional, Nutrición Clínica y Metabolismo
FGF21	fibroblast growth factor 21
GLIM	Global Leadership Initiative on Malnutrition
HIV/AIDS	human immunodeficiency virus/acquired immunodeficiency syndrome
ICU	intensive care unit
IL	interleukin
MNA	Mini Nutritional Assessment
PENSA	The Parenteral and Enteral Nutrition Society of Asia
PEM	protein-energy malnutrition
PEU	protein-energy undernutrition
(PG-) SGA	(Patient-Generated) Subjective Global Assessment
PYY	peptide YY
SASP	senescence-associated secretory phenotype
TNF-α	tumour necrosis factor alpha
WHO	World Health Organization
Zn	zinc

## References

[1] Rudnicka E., Napierała P., Podfigurna A., Męczekalski B., Smolarczyk R., Grymowicz M. (2020). The World Health Organization (WHO) approach to healthy ageing. Maturitas.

[2] United Nations (2019). World Population Prospects 2019: Highlights.

[3] Niccoli T., Partridge L. (2012). Ageing as a risk factor for disease. Curr. Biol..

[4] Biagi E., Nylund L., Candela M., Ostan R., Bucci L., Pini E., Nikkila J., Monti D., Satokari R., Franceschi C. (2010). Through ageing, and beyond: Gut microbiota and inflammatory status in seniors and centenarians. PLoS ONE.

[5] Hickson M. (2006). Malnutrition and ageing. Postgrad. Med. J..

[6] Norman K., Pichard C., Lochs H., Pirlich M. (2008). Prognostic impact of disease-related malnutrition. Clin. Nutr..

[7] Cunha A.I.L., Veronese N., de Melo Borges S., Ricci N.A. (2019). Frailty as a predictor of adverse outcomes in hospitalized older adults: A systematic review and meta-analysis. Ageing Res. Rev..

[8] Otten L., Stobäus N., Franz K., Genton L., Müller-Werdan U., Wirth R., Norman K. (2019). Impact of sarcopenia on 1-year mortality in older patients with cancer. Age Ageing.

[9] Bardon L.A., Streicher M., Corish C.A., Clarke M., Power L.C., Kenny R.A., O’Connor D.M., Laird E., O’Connor E.M., Visser M. (2020). Predictors of Incident Malnutrition in Older Irish Adults from the Irish Longitudinal Study on Ageing Cohort-A MaNuEL study. J. Gerontol. Ser. A.

[10] Dean M., Raats M.M., Grunert K.G., Lumbers M. (2009). Factors influencing eating a varied diet in old age. Public Health Nutr..

[11] Amarya S., Singh K., Sabharwal M. (2015). Changes during aging and their association with malnutrition. J. Clin. Gerontol. Geriatr..

[12] Gomes F., Schuetz P., Bounoure L., Austin P., Ballesteros-Pomar M., Cederholm T., Fletcher J., Laviano A., Norman K., Poulia K.A. (2018). ESPEN guidelines on nutritional support for polymorbid internal medicine patients. Clin. Nutr..

[13] Volkert D., Beck A.M., Cederholm T., Cereda E., Cruz-Jentoft A., Goisser S., de Groot L., Großhauser F., Kiesswetter E., Norman K. (2019). Management of Malnutrition in Older Patients-Current Approaches, Evidence and Open Questions. J. Clin. Med..

[14] Van Den Broeke C., De Burghgraeve T., Ummels M., Gescher N., Deckx L., Tjan-Heijnen V., Buntinx F., van den Akker M. (2018). Occurrence of Malnutrition and Associated Factors in Community-Dwelling Older Adults: Those with a Recent Diagnosis of Cancer Are at Higher Risk. J. Nutr. Health Aging.

[15] Eckert C., Gell N.M., Wingood M., Schollmeyer J., Tarleton E.K. (2021). Malnutrition Risk, Rurality, and Falls among Community-Dwelling Older Adults. J. Nutr. Health Aging.

[16] Khalatbari-Soltani S., Marques-Vidal P. (2015). The economic cost of hospital malnutrition in Europe; a narrative review. Clin. Nutr. ESPEN.

[17] Lengelé L., Bruyère O., Beaudart C., Reginster J.Y., Locquet M. (2021). Impact of Malnutrition Status on Muscle Parameter Changes over a 5-Year Follow-Up of Community-Dwelling Older Adults from the SarcoPhAge Cohort. Nutrients.

[18] Coin A., Sergi G., Benincà P., Lupoli L., Cinti G., Ferrara L., Benedetti G., Tomasi G., Pisent C., Enzi G. (2000). Bone mineral density and body composition in underweight and normal elderly subjects. Osteoporos. Int..

[19] Geinoz G., Rapin C.H., Rizzoli R., Kraemer R., Buchs B., Slosman D., Michel J.P., Bonjour J.P. (1993). Relationship between bone mineral density and dietary intakes in the elderly. Osteoporos. Int..

[20] Bonjour J.P., Schurch M.A., Rizzoli R. (1996). Nutritional aspects of hip fractures. Bone.

[21] Schaible U.E., Kaufmann S.H. (2007). Malnutrition and infection: Complex mechanisms and global impacts. PLoS Med..

[22] Kawakami K., Kadota J., Iida K., Shirai R., Abe K., Kohno S. (1999). Reduced immune function and malnutrition in the elderly. Tohoku J. Exp. Med..

[23] Alam I., Almajwal A., Alam W., Alam I., Ullah N., Abulmeaaty M., Razak S., Khan S., Pawelec G., Paracha P. (2019). The immune-nutrition interplay in aging facts and controversies. Nutr. Healthy Aging.

[24] Fitzpatrick F., Skally M., O’Hanlon C., Foley M., Houlihan J., Gaughan L., Smith O., Moore B., Cunneen S., Sweeney E. (2019). Food for thought. Malnutrition risk associated with increased risk of healthcare-associated infection. J. Hosp. Infect..

[25] Shpata V., Ohri I., Nurka T., Prendushi X. (2015). The prevalence and consequences of malnutrition risk in elderly Albanian intensive care unit patients. Clin. Interv. Aging.

[26] Barchitta M., Maugeri A., Favara G., Magnano San Lio R., Evola G., Agodi A., Basile G. (2019). Nutrition and Wound Healing: An Overview Focusing on the Beneficial Effects of Curcumin. Int. J. Mol. Sci..

[27] Olsson M., Järbrink K., Divakar U., Bajpai R., Upton Z., Schmidtchen A., Car J. (2019). The humanistic and economic burden of chronic wounds: A systematic review. Wound Repair Regen..

[28] Hébuterne X., Bermon S., Schneider S.M. (2001). Ageing and muscle: The effects of malnutrition, re-nutrition, and physical exercise. Curr. Opin. Clin. Nutr. Metab. Care.

[29] Inouye S.K., Studenski S., Tinetti M.E., Kuchel G.A. (2007). Geriatric syndromes: Clinical, research, and policy implications of a core geriatric concept. J. Am. Geriatr. Soc..

[30] Kane R.L., Shamliyan T., Talley K., Pacala J. (2012). The association between geriatric syndromes and survival. J. Am. Geriatr. Soc..

[31] Rausch C., van Zon S.K.R., Liang Y., Laflamme L., Möller J., de Rooij S.E., Bültmann U. (2021). Geriatric Syndromes and Incident Chronic Health Conditions Among 9094 Older Community-Dwellers: Findings from the Lifelines Cohort Study. J. Am. Med. Dir. Assoc..

[32] Won C.W., Yoo H.J., Yu S.H., Kim C.O., Dumlao L.C.I., Dewiasty E., Rowland J., Chang H.H., Wang J., Akishita M. (2013). Lists of geriatric syndromes in the Asian-Pacific geriatric societies. Eur. Geriatr. Med..

[33] Tan V.M.H., Pang B.W.J., Lau L.K., Jabbar K.A., Seah W.T., Chen K.K., Ng T.P., Wee S.L. (2021). Malnutrition and Sarcopenia in Community-Dwelling Adults in Singapore: Yishun Health Study. J. Nutr. Health Aging.

[34] Juby A.G., Mager D.R. (2019). A review of nutrition screening tools used to assess the malnutrition-sarcopenia syndrome (MSS) in the older adult. Clin. Nutr. ESPEN.

[35] Stenholm S., Harris T.B., Rantanen T., Visser M., Kritchevsky S.B., Ferrucci L. (2008). Sarcopenic obesity: Definition, cause and consequences. Curr. Opin. Clin. Nutr. Metab. Care.

[36] Fougère B., Morley J.E. (2017). Editorial: Weight Loss is a Major Cause of Frailty. J. Nutr. Health Aging.

[37] Boulos C., Salameh P., Barberger-Gateau P. (2016). Malnutrition and frailty in community dwelling older adults living in a rural setting. Clin. Nutr..

[38] Jeejeebhoy K.N. (2012). Malnutrition, fatigue, frailty, vulnerability, sarcopenia and cachexia: Overlap of clinical features. Curr. Opin. Clin. Nutr. Metab. Care.

[39] Gingrich A., Volkert D., Kiesswetter E., Thomanek M., Bach S., Sieber C.C., Zopf Y. (2019). Prevalence and overlap of sarcopenia, frailty, cachexia and malnutrition in older medical inpatients. BMC Geriatr..

[40] Norazman C.W., Adznam S.N., Jamaluddin R. (2020). Malnutrition as Key Predictor of Physical Frailty among Malaysian Older Adults. Nutrients.

[41] Norazman C.W., Adznam S.N., Jamaluddin R. (2020). Physical Frailty among Urban-Living Community-Dwelling Older Adults in Malaysia. Int. J. Environ. Res. Public Health.

[42] Wei K., Nyunt M.S., Gao Q., Wee S.L., Yap K.B., Ng T.P. (2018). Association of Frailty and Malnutrition With Long-term Functional and Mortality Outcomes Among Community-Dwelling Older Adults: Results From the Singapore Longitudinal Aging Study 1. JAMA Netw. Open.

[43] Zengarini E., Ruggiero C., Pérez-Zepeda M.U., Hoogendijk E.O., Vellas B., Mecocci P., Cesari M. (2015). Fatigue: Relevance and implications in the aging population. Exp. Gerontol..

[44] Filler K., Lyon D., Bennett J., McCain N., Elswick R., Lukkahatai N., Saligan L.N. (2014). Association of Mitochondrial Dysfunction and Fatigue: A Review of the Literature. BBA Clin..

[45] Herpich C., Franz K., Klaus S., Müller-Werdan U., Ost M., Norman K. (2021). Age-related fatigue is associated with reduced mitochondrial function in peripheral blood mononuclear cells. Exp. Gerontol..

[46] Franz K., Otten L., Müller-Werdan U., Doehner W., Norman K. (2019). Severe Weight Loss and Its Association with Fatigue in Old Patients at Discharge from a Geriatric Hospital. Nutrients.

[47] Azzolino D., Arosio B., Marzetti E., Calvani R., Cesari M. (2020). Nutritional Status as a Mediator of Fatigue and Its Underlying Mechanisms in Older People. Nutrients.

[48] Haß U., Herpich C., Norman K. (2019). Anti-Inflammatory Diets and Fatigue. Nutrients.

[49] Ogawa S. (2014). Nutritional management of older adults with cognitive decline and dementia. Geriatr. Gerontol. Int..

[50] Yu W., Yu W., Liu X., Wan T., Chen C., Xiong L., Zhang W., Lü Y. (2021). Associations between malnutrition and cognitive impairment in an elderly Chinese population: An analysis based on a 7-year database. Psychogeriatrics.

[51] Cabrera M.A., Mesas A.E., Garcia A.R., de Andrade S.M. (2007). Malnutrition and depression among community-dwelling elderly people. J. Am. Med. Dir. Assoc..

[52] Smoliner C., Norman K., Wagner K.H., Hartig W., Lochs H., Pirlich M. (2009). Malnutrition and depression in the institutionalised elderly. Br. J. Nutr..

[53] Yoshimura K., Yamada M., Kajiwara Y., Nishiguchi S., Aoyama T. (2013). Relationship between depression and risk of malnutrition among community-dwelling young-old and old-old elderly people. Aging Ment. Health.

[54] Gentile S., Lacroix O., Durand A.C., Cretel E., Alazia M., Sambuc R., Bonin-Guillaume S. (2013). Malnutrition: A highly predictive risk factor of short-term mortality in elderly presenting to the emergency department. J. Nutr. Health Aging.

[55] Komici K., Vitale D.F., Mancini A., Bencivenga L., Conte M., Provenzano S., Grieco F.V., Visaggi L., Ronga I., Cittadini A. (2019). Impact of Malnutrition on Long-Term Mortality in Elderly Patients with Acute Myocardial Infarction. Nutrients.

[56] Söderström L., Rosenblad A., Thors Adolfsson E., Bergkvist L. (2017). Malnutrition is associated with increased mortality in older adults regardless of the cause of death. Br. J. Nutr..

[57] Teigen L.M., Kuchnia A.J., Nagel E.M., Price K.L., Hurt R.T., Earthman C.P. (2018). Diagnosing clinical malnutrition: Perspectives from the past and implications for the future. Clin. Nutr. ESPEN.

[58] Cederholm T., Jensen G.L., Correia M., Gonzalez M.C., Fukushima R., Higashiguchi T., Baptista G., Barazzoni R., Blaauw R., Coats A. (2019). GLIM criteria for the diagnosis of malnutrition–A consensus report from the global clinical nutrition community. Clin. Nutr..

[59] De van der Schueren M.A.E., Keller H., Cederholm T., Barazzoni R., Compher C., Correia M., Gonzalez M.C., Jager-Wittenaar H., Pirlich M., Steiber A. (2020). Global Leadership Initiative on Malnutrition (GLIM): Guidance on validation of the operational criteria for the diagnosis of protein-energy malnutrition in adults. Clin. Nutr..

[60] Yeung S.S.Y., Chan R.S.M., Kwok T., Lee J.S.W., Woo J. (2020). Malnutrition According to GLIM Criteria and Adverse Outcomes in Community-Dwelling Chinese Older Adults: A Prospective Analysis. J. Am. Med. Dir. Assoc..

[61] Cederholm T., Barazzoni R., Austin P., Ballmer P., Biolo G., Bischoff S.C., Compher C., Correia I., Higashiguchi T., Holst M. (2017). ESPEN guidelines on definitions and terminology of clinical nutrition. Clin. Nutr..

[62] Cereda E. (2012). Mini nutritional assessment. Curr. Opin. Clin. Nutr. Metab. Care.

[63] Vandewoude M.F., Alish C.J., Sauer A.C., Hegazi R.A. (2012). Malnutrition-sarcopenia syndrome: Is this the future of nutrition screening and assessment for older adults?. J. Aging Res..

[64] Kinosian B., Jeejeebhoy K.N. (1995). What is malnutrition? Does it matter?. Nutrition.

[65] Bauer J., Biolo G., Cederholm T., Cesari M., Cruz-Jentoft A.J., Morley J.E., Phillips S., Sieber C., Stehle P., Teta D. (2013). Evidence-based recommendations for optimal dietary protein intake in older people: A position paper from the PROT-AGE Study Group. J. Am. Med. Dir. Assoc..

[66] Deutz N.E., Bauer J.M., Barazzoni R., Biolo G., Boirie Y., Bosy-Westphal A., Cederholm T., Cruz-Jentoft A., Krznaric Z., Nair K.S. (2014). Protein intake and exercise for optimal muscle function with aging: Recommendations from the ESPEN Expert Group. Clin. Nutr..

[67] Hengeveld L.M., Wijnhoven H.A.H., Olthof M.R., Brouwer I.A., Harris T.B., Kritchevsky S.B., Newman A.B., Visser M. (2018). Prospective associations of poor diet quality with long-term incidence of protein-energy malnutrition in community-dwelling older adults: The Health, Aging, and Body Composition (Health ABC) Study. Am. J. Clin. Nutr..

[68] Hengeveld L.M., Boer J.M.A., Gaudreau P., Heymans M.W., Jagger C., Mendonça N., Ocké M.C., Presse N., Sette S., Simonsick E.M. (2020). Prevalence of protein intake below recommended in community-dwelling older adults: A meta-analysis across cohorts from the PROMISS consortium. J. Cachexia Sarcopenia Muscle.

[69] Conzade R., Koenig W., Heier M., Schneider A., Grill E., Peters A., Thorand B. (2017). Prevalence and Predictors of Subclinical Micronutrient Deficiency in German Older Adults: Results from the Population-Based KORA-Age Study. Nutrients.

[70] IOM (Institute of Medicine) (2010). Providing Healthy and Safe Foods as We Age: Workshop Summary.

[71] Hamer D.H., Sempértegui F., Estrella B., Tucker K.L., Rodríguez A., Egas J., Dallal G.E., Selhub J., Griffiths J.K., Meydani S.N. (2009). Micronutrient deficiencies are associated with impaired immune response and higher burden of respiratory infections in elderly Ecuadorians. J. Nutr..

[72] Wallace T.C., Frankenfeld C.L., Frei B., Shah A.V., Yu C.R., van Klinken B.J., Adeleke M. (2019). Multivitamin/Multimineral Supplement Use is Associated with Increased Micronutrient Intakes and Biomarkers and Decreased Prevalence of Inadequacies and Deficiencies in Middle-Aged and Older Adults in the United States. J. Nutr. Gerontol. Geriatr..

[73] Elmadfa I., Meyer A.L., Marriott B.P., Birt D.F., Stallings V.A., Yates A.A. (2020). Present Knowledge in Nutrition. Chapter 5–Nutrition, Aging, and Requirements in the Elderly.

[74] Maggini S., Pierre A., Calder P.C. (2018). Immune Function and Micronutrient Requirements Change over the Life Course. Nutrients.

[75] Karadima V., Kraniotou C., Bellos G., Tsangaris G.T. (2016). Drug-micronutrient interactions: Food for thought and thought for action. EPMA J..

[76] Gröber U., Schmidt J., Kisters K. (2020). Important drug-micronutrient interactions: A selection for clinical practice. Crit. Rev. Food Sci. Nutr..

[77] Kehl-Fie T.E., Skaar E.P. (2010). Nutritional immunity beyond iron: A role for manganese and zinc. Curr. Opin. Chem. Biol..

[78] Mocchegiani E., Romeo J., Malavolta M., Costarelli L., Giacconi R., Diaz L.E., Marcos A. (2013). Zinc: Dietary intake and impact of supplementation on immune function in elderly. Age.

[79] Jung A., Spira D., Steinhagen-Thiessen E., Demuth I., Norman K. (2017). Zinc Deficiency Is associated With Depressive Symptoms-Results From the Berlin Aging Study II. J. Gerontol. Ser. A Biol. Sci. Med. Sci..

[80] Suzuki H., Asakawa A., Li J.B., Tsai M., Amitani H., Ohinata K., Komai M., Inui A. (2011). Zinc as an appetite stimulator–the possible role of zinc in the progression of diseases such as cachexia and sarcopenia. Recent Pat. Food Nutr. Agric..

[81] Klotz L.O., Kröncke K.D., Buchczyk D.P., Sies H. (2003). Role of copper, zinc, selenium and tellurium in the cellular defense against oxidative and nitrosative stress. J. Nutr..

[82] Mocchegiani E., Malavolta M. (2008). Zinc-gene interaction related to inflammatory/immune response in ageing. Genes Nutr..

[83] Mocchegiani E., Basso A., Giacconi R., Piacenza F., Costarelli L., Pierpaoli S., Malavolta M. (2010). Diet (zinc)-gene interaction related to inflammatory/immune response in ageing: Possible link with frailty syndrome?. Biogerontology.

[84] Lauretani F., Semba R.D., Bandinelli S., Dayhoff-Brannigan M., Giacomini V., Corsi A.M., Guralnik J.M., Ferrucci L. (2008). Low plasma carotenoids and skeletal muscle strength decline over 6 years. J. Gerontol. Ser. A Biol. Sci. Med. Sci..

[85] Lauretani F., Semba R.D., Bandinelli S., Dayhoff-Brannigan M., Lauretani F., Corsi A.M., Guralnik J.M., Ferrucci L. (2008). Carotenoids as protection against disability in older persons. Rejuvenation Res..

[86] Bartali B., Frongillo E.A., Guralnik J.M., Stipanuk M.H., Allore H.G., Cherubini A., Bandinelli S., Ferrucci L., Gill T.M. (2008). Serum micronutrient concentrations and decline in physical function among older persons. JAMA.

[87] Cesari M., Pahor M., Bartali B., Cherubini A., Penninx B.W., Williams G.R., Atkinson H., Martin A., Guralnik J.M., Ferrucci L. (2004). Antioxidants and physical performance in elderly persons: The Invecchiare in Chianti (InCHIANTI) study. Am. J. Clin. Nutr..

[88] Sánchez-Rodríguez D., Annweiler C., Ronquillo-Moreno N., Tortosa-Rodríguez A., Guillén-Solà A., Vázquez-Ibar O., Escalada F., Muniesa J.M., Marco E. (2018). Clinical application of the basic definition of malnutrition proposed by the European Society for Clinical Nutrition and Metabolism (ESPEN): Comparison with classical tools in geriatric care. Arch. Gerontol. Geriatr..

[89] Leij-Halfwerk S., Verwijs M.H., van Houdt S., Borkent J.W., Guaitoli P.R., Pelgrim T., Heymans M.W., Power L., Visser M., Corish C.A. (2019). Prevalence of protein-energy malnutrition risk in European older adults in community, residential and hospital settings, according to 22 malnutrition screening tools validated for use in adults >/=65 years: A systematic review and meta-analysis. Maturitas.

[90] Crichton M., Craven D., Mackay H., Marx W., de van der Schueren M., Marshall S. (2019). A systematic review, meta-analysis and meta-regression of the prevalence of protein-energy malnutrition: Associations with geographical region and sex. Age Ageing.

[91] Cereda E., Pedrolli C., Klersy C., Bonardi C., Quarleri L., Cappello S., Turri A., Rondanelli M., Caccialanza R. (2016). Nutritional status in older persons according to healthcare setting: A systematic review and meta-analysis of prevalence data using MNA^®^. Clin. Nutr..

[92] Wolters M., Volkert D., Streicher M., Kiesswetter E., Torbahn G., O’Connor E.M., O’Keeffe M., Kelly M., O’Herlihy E., O’Toole P.W. (2019). Prevalence of malnutrition using harmonized definitions in older adults from different settings–A MaNuEL study. Clin. Nutr..

[93] Streicher M., van Zwienen-Pot J., Bardon L., Nagel G., Teh R., Meisinger C., Colombo M., Torbahn G., Kiesswetter E., Flechtner-Mors M. (2018). Determinants of Incident Malnutrition in Community-Dwelling Older Adults: A MaNuEL Multicohort Meta-Analysis. J. Am. Geriatr. Soc..

[94] O’Keeffe M., Kelly M., O’Herlihy E., O’Toole P.W., Kearney P.M., Timmons S., O’Shea E., Stanton C., Hickson M., Rolland Y. (2019). Potentially modifiable determinants of malnutrition in older adults: A systematic review. Clin. Nutr..

[95] Volkert D., Kiesswetter E., Cederholm T., Donini L.M., Eglseer D., Norman K., Schneider S.M., Ströbele-Benschop N., Torbahn G., Wirth R. (2019). Development of a Model on Determinants of Malnutrition in Aged Persons: A MaNuEL Project. Gerontol. Geriatr. Med..

[96] Rémond D., Shahar D.R., Gille D., Pinto P., Kachal J., Peyron M.A., Dos Santos C.N., Walther B., Bordoni A., Dupont D. (2015). Understanding the gastrointestinal tract of the elderly to develop dietary solutions that prevent malnutrition. Oncotarget.

[97] An R., Wilms E., Masclee A.A.M., Smidt H., Zoetendal E.G., Jonkers D. (2018). Age-dependent changes in GI physiology and microbiota: Time to reconsider?. Gut.

[98] Nagpal R., Mainali R., Ahmadi S., Wang S., Singh R., Kavanagh K., Kitzman D.W., Kushugulova A., Marotta F., Yadav H. (2018). Gut microbiome and aging: Physiological and mechanistic insights. Nutr. Healthy Aging.

[99] Van de Wouw M., Schellekens H., Dinan T.G., Cryan J.F. (2017). Microbiota-Gut-Brain Axis: Modulator of Host Metabolism and Appetite. J. Nutr..

[100] Ticinesi A., Lauretani F., Milani C., Nouvenne A., Tana C., Del Rio D., Maggio M., Ventura M., Meschi T. (2017). Aging Gut Microbiota at the Cross-Road between Nutrition, Physical Frailty, and Sarcopenia: Is There a Gut-Muscle Axis?. Nutrients.

[101] Moss C., Dhillo W.S., Frost G., Hickson M. (2012). Gastrointestinal hormones: The regulation of appetite and the anorexia of ageing. J. Hum. Nutr. Diet..

[102] Atalayer D., Astbury N.M. (2013). Anorexia of aging and gut hormones. Aging Dis..

[103] Morley J.E., Miller D.K., Perry H.M., Patrick P., Guigoz Y., Vellas B. (1999). Anorexia of aging, leptin, and the Mini Nutritional Assessment. Nestle Nutr. Workshop Ser. Clin. Perform. Programme.

[104] Ueno H., Nakazato M. (2016). Mechanistic relationship between the vagal afferent pathway, central nervous system and peripheral organs in appetite regulation. J. Diabetes Investig..

[105] Kirsz K., Zieba D.A. (2011). Ghrelin-mediated appetite regulation in the central nervous system. Peptides.

[106] Pliquett R.U., Führer D., Falk S., Zysset S., von Cramon D.Y., Stumvoll M. (2006). The effects of insulin on the central nervous system--focus on appetite regulation. Horm. Metab. Res..

[107] Serra-Prat M., Mans E., Palomera E., Clavé P. (2013). Gastrointestinal peptides, gastrointestinal motility, and anorexia of aging in frail elderly persons. Neurogastroenterol. Motil..

[108] Bauer J.M., Haack A., Winning K., Wirth R., Fischer B., Uter W., Erdmann J., Schusdziarra V., Sieber C.C. (2010). Impaired postprandial response of active ghrelin and prolonged suppression of hunger sensation in the elderly. J. Gerontol. Ser. A Biol. Sci. Med. Sci..

[109] Morton G.J., Cummings D.E., Baskin D.G., Barsh G.S., Schwartz M.W. (2006). Central nervous system control of food intake and body weight. Nature.

[110] Brown W.E., Bradford B.J. (2021). Invited review: Mechanisms of hypophagia during disease. J. Dairy Sci..

[111] Wysokinski A., Sobow T., Kloszewska I., Kostka T. (2015). Mechanisms of the anorexia of aging—A review. Age.

[112] Cox N.J., Ibrahim K., Sayer A.A., Robinson S.M., Roberts H.C. (2019). Assessment and Treatment of the Anorexia of Aging: A Systematic Review. Nutrients.

[113] Giezenaar C., Chapman I., Luscombe-Marsh N., Feinle-Bisset C., Horowitz M., Soenen S. (2016). Ageing Is Associated with Decreases in Appetite and Energy Intake--A Meta-Analysis in Healthy Adults. Nutrients.

[114] Roy M., Shatenstein B., Gaudreau P., Morais J.A., Payette H. (2015). Seniors’ body weight dissatisfaction and longitudinal associations with weight changes, anorexia of aging, and obesity: Results from the NuAge Study. J. Aging Health.

[115] Morley J.E. (2012). Anorexia of aging: A true geriatric syndrome. J. Nutr. Health Aging.

[116] Landi F., Liperoti R., Russo A., Giovannini S., Tosato M., Barillaro C., Capoluongo E., Bernabei R., Onder G. (2013). Association of anorexia with sarcopenia in a community-dwelling elderly population: Results from the ilSIRENTE study. Eur. J. Nutr..

[117] Tsutsumimoto K., Doi T., Makizako H., Hotta R., Nakakubo S., Makino K., Suzuki T., Shimada H. (2017). The association between anorexia of aging and physical frailty: Results from the national center for geriatrics and gerontology’s study of geriatric syndromes. Maturitas.

[118] Wall B.T., Gorissen S.H., Pennings B., Koopman R., Groen B.B., Verdijk L.B., van Loon L.J. (2015). Aging Is Accompanied by a Blunted Muscle Protein Synthetic Response to Protein Ingestion. PLoS ONE.

[119] Dickinson J.M., Volpi E., Rasmussen B.B. (2013). Exercise and nutrition to target protein synthesis impairments in aging skeletal muscle. Exerc. Sport Sci. Rev..

[120] Drummond M.J., Dreyer H.C., Pennings B., Fry C.S., Dhanani S., Dillon E.L., Sheffield-Moore M., Volpi E., Rasmussen B.B. (2008). Skeletal muscle protein anabolic response to resistance exercise and essential amino acids is delayed with aging. J. Appl. Physiol..

[121] Markofski M.M., Dickinson J.M., Drummond M.J., Fry C.S., Fujita S., Gundermann D.M., Glynn E.L., Jennings K., Paddon-Jones D., Reidy P.T. (2015). Effect of age on basal muscle protein synthesis and mTORC1 signaling in a large cohort of young and older men and women. Exp. Gerontol..

[122] Morais J.A., Jacob K.W., Chevalier S. (2018). Effects of aging and insulin resistant states on protein anabolic responses in older adults. Exp. Gerontol..

[123] Barclay R.D., Burd N.A., Tyler C., Tillin N.A., Mackenzie R.W. (2019). The Role of the IGF-1 Signaling Cascade in Muscle Protein Synthesis and Anabolic Resistance in Aging Skeletal Muscle. Front. Nutr..

[124] Basu R., Dalla Man C., Campioni M., Basu A., Klee G., Toffolo G., Cobelli C., Rizza R.A. (2006). Effects of age and sex on postprandial glucose metabolism: Differences in glucose turnover, insulin secretion, insulin action, and hepatic insulin extraction. Diabetes.

[125] Herpich C., Haß U., Kochlik B., Franz K., Laeger T., Klaus S., Bosy-Westphal A., Norman K. (2021). Postprandial dynamics and response of fibroblast growth factor 21 in older adults. Clin. Nutr..

[126] Conte M., Ostan R., Fabbri C., Santoro A., Guidarelli G., Vitale G., Mari D., Sevini F., Capri M., Sandri M. (2018). Human aging and longevity are characterized by high levels of mitokines. J. Gerontol. Ser. A Biol. Sci. Med. Sci..

[127] Oost L.J., Kustermann M., Armani A., Blaauw B., Romanello V. (2019). Fibroblast growth factor 21 controls mitophagy and muscle mass. J. Cachexia Sarcopenia Muscle.

[128] Tezze C., Romanello V., Desbats M.A., Fadini G.P., Albiero M., Favaro G., Ciciliot S., Soriano M.E., Morbidoni V., Cerqua C. (2017). Age-Associated Loss of OPA1 in Muscle Impacts Muscle Mass, Metabolic Homeostasis, Systemic Inflammation, and Epithelial Senescence. Cell Metab..

[129] Sun H., Sherrier M., Li H. (2021). Skeletal Muscle and Bone–Emerging Targets of Fibroblast Growth Factor-21. Front. Physiol..

[130] Franz K., Ost M., Otten L., Herpich C., Coleman V., Endres A.S., Klaus S., Müller-Werdan U., Norman K. (2019). Higher serum levels of fibroblast growth factor 21 in old patients with cachexia. Nutrition.

[131] Lin Z., Tian H., Lam K.S., Lin S., Hoo R.C., Konishi M., Itoh N., Wang Y., Bornstein S.R., Xu A. (2013). Adiponectin mediates the metabolic effects of FGF21 on glucose homeostasis and insulin sensitivity in mice. Cell Metab..

[132] Menzaghi C., Trischitta V. (2018). The Adiponectin Paradox for All-Cause and Cardiovascular Mortality. Diabetes.

[133] Herpich C., Kochlik B., Haß U., Weber D., Grune T., Norman K. (2021). Altered Adiponectin Response in Older Women Following Dextrose and High-Fat Dietary Challenges. Mol. Nutr. Food Res..

[134] Franceschi C., Bonafè M., Valensin S., Olivieri F., De Luca M., Ottaviani E., De Benedictis G. (2000). Inflamm-aging. An evolutionary perspective on immunosenescence. Ann. N. Y. Acad. Sci..

[135] Meftahi G.H., Jangravi Z., Sahraei H., Bahari Z. (2020). The possible pathophysiology mechanism of cytokine storm in elderly adults with COVID-19 infection: The contribution of “inflame-aging”. Inflamm. Res..

[136] Omarjee L., Perrot F., Meilhac O., Mahe G., Bousquet G., Janin A. (2020). Immunometabolism at the cornerstone of inflammaging, immunosenescence, and autoimmunity in COVID-19. Aging.

[137] Rothenberg E. (2021). Coronavirus Disease 19 from the Perspective of Ageing with Focus on Nutritional Status and Nutrition Management-A Narrative Review. Nutrients.

[138] Morrisette-Thomas V., Cohen A.A., Fulop T., Riesco E., Legault V., Li Q., Milot E., Dusseault-Belanger F., Ferrucci L. (2014). Inflamm-aging does not simply reflect increases in pro-inflammatory markers. Mech. Ageing Dev..

[139] Minciullo P.L., Catalano A., Mandraffino G., Casciaro M., Crucitti A., Maltese G., Morabito N., Lasco A., Gangemi S., Basile G. (2016). Inflammaging and Anti-Inflammaging: The Role of Cytokines in Extreme Longevity. Arch. Immunol. Ther. Exp..

[140] Pinti M., Cevenini E., Nasi M., De Biasi S., Salvioli S., Monti D., Benatti S., Gibellini L., Cotichini R., Stazi M.A. (2014). Circulating mitochondrial DNA increases with age and is a familiar trait: Implications for “inflamm-aging”. Eur. J. Immunol..

[141] Conte M., Martucci M., Chiariello A., Franceschi C., Salvioli S. (2020). Mitochondria, immunosenescence and inflammaging: A role for mitokines?. Seminars in Immunopathology.

[142] Onyango I.G., Jauregui G.V., Čarná M., Bennett J.P., Stokin G.B. (2021). Neuroinflammation in Alzheimer’s Disease. Biomedicines.

[143] Liberale L., Montecucco F., Tardif J.C., Libby P., Camici G.G. (2020). Inflamm-ageing: The role of inflammation in age-dependent cardiovascular disease. Eur. Heart J..

[144] Livshits G., Kalinkovich A. (2019). Inflammaging as a common ground for the development and maintenance of sarcopenia, obesity, cardiomyopathy and dysbiosis. Ageing Res. Rev..

[145] Prattichizzo F., De Nigris V., Spiga R., Mancuso E., La Sala L., Antonicelli R., Testa R., Procopio A.D., Olivieri F., Ceriello A. (2018). Inflammageing and metaflammation: The yin and yang of type 2 diabetes. Ageing Res. Rev..

[146] Kirk B., Feehan J., Lombardi G., Duque G. (2020). Muscle, Bone, and Fat Crosstalk: The Biological Role of Myokines, Osteokines, and Adipokines. Curr. Osteoporos. Rep..

[147] Marzetti E., Picca A., Marini F., Biancolillo A., Coelho-Junior H.J., Gervasoni J., Bossola M., Cesari M., Onder G., Landi F. (2019). Inflammatory signatures in older persons with physical frailty and sarcopenia: The frailty “cytokinome” at its core. Exp. Gerontol..

[148] Ostan R., Lanzarini C., Pini E., Scurti M., Vianello D., Bertarelli C., Fabbri C., Izzi M., Palmas G., Biondi F. (2015). Inflammaging and cancer: A challenge for the Mediterranean diet. Nutrients.

[149] Olivieri F., Rippo M.R., Monsurrò V., Salvioli S., Capri M., Procopio A.D., Franceschi C. (2013). MicroRNAs linking inflamm-aging, cellular senescence and cancer. Ageing Res. Rev..

[150] Bonafè M., Storci G., Franceschi C. (2012). Inflamm-aging of the stem cell niche: Breast cancer as a paradigmatic example: Breakdown of the multi-shell cytokine network fuels cancer in aged people. Bioessays.

[151] Calder P.C., Bosco N., Bourdet-Sicard R., Capuron L., Delzenne N., Dore J., Franceschi C., Lehtinen M.J., Recker T., Salvioli S. (2017). Health relevance of the modification of low grade inflammation in ageing (inflammageing) and the role of nutrition. Ageing Res. Rev..

[152] Yeh S.S., Schuster M.W. (1999). Geriatric cachexia: The role of cytokines. Am. J. Clin. Nutr..

[153] Evans W.J., Morley J.E., Argiles J., Bales C., Baracos V., Guttridge D., Jatoi A., Kalantar-Zadeh K., Lochs H., Mantovani G. (2008). Cachexia: A new definition. Clin. Nutr..

[154] Tracey K.J., Wei H., Manogue K.R., Fong Y., Hesse D.G., Nguyen H.T., Kuo G.C., Beutler B., Cotran R.S., Cerami A. (1988). Cachectin/tumor necrosis factor induces cachexia, anemia, and inflammation. J. Exp. Med..

[155] Oldenburg H.S., Rogy M.A., Lazarus D.D., Van Zee K.J., Keeler B.P., Chizzonite R.A., Lowry S.F., Moldawer L.L. (1993). Cachexia and the acute-phase protein response in inflammation are regulated by interleukin-6. Eur. J. Immunol..

[156] Ershler W.B. (1993). Interleukin-6: A cytokine for gerontologists. J. Am. Geriatr. Soc..

[157] Boirie Y. (2009). Physiopathological mechanism of sarcopenia. J. Nutr. Health Aging.

[158] Fatyga P., Pac A., Fedyk-Łukasik M., Grodzicki T., Skalska A. (2020). The relationship between malnutrition risk and inflammatory biomarkers in outpatient geriatric population. Eur. Geriatr. Med..

[159] Correa-Pérez A., Abraha I., Cherubini A., Collinson A., Dardevet D., de Groot L.C.P.G.M. (2019). Efficacy of non-pharmacological interventions on nutritional status and clinical outcomes in older people with malnutrition or at risk of malnutrition: A systematic review. The SENATOR project ONTOP series and MaNuEL knowledge hub project. Aging Res. Rev..

[160] Reinders I., Volkert D., de Groot L., Beck A.M., Feldblum I., Jobse I., Neelemaat F., de van der Schueren M.A.E., Shahar D.R., Smeets E. (2019). Effectiveness of nutritional interventions in older adults at risk of malnutrition across different health care settings: Pooled analyses of individual participant data from nine randomized controlled trials. Clin. Nutr..

[161] Höhn A., Weber D., Jung T., Ott C., Hugo M., Kochlik B., Kehm R., König J., Grune T., Castro J.P. (2017). Happily (n)ever after: Aging in the context of oxidative stress, proteostasis loss and cellular senescence. Redox Biol..

[162] Fisher L. (2019). Food Science and Nutrition.

[163] Ghosh T.S., Rampelli S., Jeffery I.B., Santoro A., Neto M., Capri M., Giampieri E., Jennings A., Candela M., Turroni S. (2020). Mediterranean diet intervention alters the gut microbiome in older people reducing frailty and improving health status: The NU-AGE 1-year dietary intervention across five European countries. Gut.

[164] Deutz N.E.P., Ashurst I., Ballesteros M.D., Bear D.E., Cruz-Jentoft A.J., Genton L., Landi F., Laviano A., Norman K., Prado C.M. (2019). The Underappreciated Role of Low Muscle Mass in the Management of Malnutrition. J. Am. Med. Dir. Assoc..

[165] Van der Pols-Vijlbrief R., Wijnhoven H.A.H., Bosmans J.E., Twisk J.W.R., Visser M. (2017). Targeting the underlying causes of undernutrition. Cost-effectiveness of a multifactorial personalized intervention in community-dwelling older adults: A randomized controlled trial. Clin. Nutr..

